# Effect of Three Weeks of High-Intensity, Long-Term Preoperative Rehabilitation for Esophageal Cancer Patients with Stroke Sequelae Who Were Considered Unfit for Surgery Due to Low Activity: A Case Report

**DOI:** 10.3390/healthcare11050665

**Published:** 2023-02-24

**Authors:** Tokio Kinoshita, Yukihide Nishimura, Rikito Zaiki, Yoshinori Yasuoka, Yasunori Umemoto, Yumi Koike, Makoto Kawanishi, Fumihiro Tajima

**Affiliations:** 1 Department of Rehabilitation Medicine, Wakayama Medical University, Wakayama 641-8509, Japan; 2 Division of Rehabilitation, Wakayama Medical University Hospital, Wakayama 641-8510, Japan; 3 Department of Rehabilitation Medicine, Iwate Medical University, Shiwa-gun 028-3639, Japan

**Keywords:** esophageal cancer, preoperative rehabilitation, postoperative complications, performance status, chronic cerebrovascular disease

## Abstract

Treatment of esophageal cancer is based on tumor-node-metastasis (TNM) classification, and surgical treatment is chosen based on the patient’s ability to tolerate surgery. Surgical endurance partly depends on activity status, with performance status (PS) generally used as an indicator. This report describes a 72-year-old man with lower esophageal cancer and an 8-year history of severe left hemiplegia. He had sequelae of cerebral infarction and a TNM classification of T3, N1, and M0, and was judged ineligible for surgery because his PS was grade three; he underwent preoperative rehabilitation with hospitalization for 3 weeks. He had been able to walk with a cane in the past, but once he was diagnosed with esophageal cancer, he began using a wheelchair and was dependent on assistance from his family in his daily life. Rehabilitation consisted of strength training, aerobic exercise, gait training, and activities of daily living (ADL) training for 5 h a day, according to the patient’s condition. After 3 weeks of rehabilitation, his ADL ability and PS improved sufficiently for surgical indication. No complications occurred postoperatively, and he was discharged when his ADL ability was higher than that before preoperative rehabilitation. This case provides valuable information for the rehabilitation of patients with inactive esophageal cancer.

## 1. Introduction

Treatment plans for patients with esophageal cancer are determined in accordance with the Japanese guidelines based on tumor-node-metastasis (TNM) classification [1]. In cases of category 3 tumors of the TNM classification, the indication for a treatment plan is determined based on the presence or absence of surgical tolerance in our hospital. Although a clear standard for surgical tolerance has not been established, it is determined based on activity status in addition to cardiopulmonary, renal, liver function, and glucose tolerance. Performance status (PS) is a commonly used index for evaluating activity status [2], and PS 0–2 (the patient is ambulatory and capable of self-care but unable to perform work activities and is active more than 50% of waking hours) is considered desirable for surgical indications, in terms of decreasing the occurrence of postoperative complications [2]. However, if the amount of activity is lower than that of PS 3 (capable of only limited self-care and confined to a bed or chair for more than 50% of waking hours), chemoradiotherapy and palliative symptomatic therapy are often chosen instead of surgery (Table 1).

The reported incidence of postoperative pulmonary complications associated with esophagectomy ranges from 30–60% [3,4], and has been shown to increase mortality, prolong hospital stay, and present additional medical costs [5]. While numerous studies have shown that preoperative rehabilitation helps prevent postoperative complications [6,7,8,9,10], it is also important for improving and maintaining patients’ activities of daily living (ADL) and activity before deciding on a treatment plan and proceeding with the treatment.

Patients with cerebrovascular disorders suffer from a wide range of sequelae, including higher brain dysfunction, motor paralysis, and sensory impairment, depending on the site of brain damage [11]. This results in a long-term decline in ADL ability and activity [12]. Preoperative rehabilitation for patients who are not eligible for surgery due to low activity tolerance, or for patients with esophageal cancer complicated by cerebrovascular disease, has not yet been reported.

This case report describes a patient with esophageal cancer with sequelae of cerebral infarction and a TNM classification of T3, N1, and M0, who was determined to not be eligible for surgery because his PS was 3. Instead, he underwent preoperative rehabilitation with hospitalization for 3 weeks. This is a detailed description of the patient’s rehabilitation until his PS improved and surgery became possible, as well as the course of his rehabilitation until discharge after surgery.

## 2. Case Report

The patient was a 72-year-old man with esophageal cancer. He visited his primary care physician complaining of esophageal transit disturbance, and was diagnosed with lower esophageal cancer by upper gastrointestinal endoscopy and computed tomography (TNM classification: T3, N1, M0). The patient had a cerebral infarction 8 years prior, with left hemiplegia as an aftereffect. However, he was able to walk independently with the use of a cane and could perform ADLs independently. After being discharged from the hospital, the patient did not undergo outpatient rehabilitation or exercise on his own in order to prevent the deterioration of physical function. Thereafter, his activity gradually declined with age, and at the time of his diagnosis of esophageal cancer, he used a gait aid and a lower limb brace to walk around at home and a wheelchair for outdoor mobility. The patient was not in the habit of performing exercise and spent > 50% of the day in bed. The patient’s PS was 3 at the time of diagnosis, and after a comprehensive review of his operative tolerance, surgery was not indicated. However, the gastroenterological surgeon consulted with the Department of Rehabilitation about whether PS could be improved through rehabilitation, and the Department of Rehabilitation began to consider it.

At the time of the examination in the Department of Rehabilitation, the Glasgow Coma scale was E4V5M6 [13,14] and the patient’s verbal communication was good. A cranial nerve examination revealed mild drooping of the left angle of the mouth due to facial nerve palsy. The National Institutes of Health Stroke Scale results were as follows: 1 point for “Level of consciousness commands,” 4 points for “Motor arm”, 3 points for “Motor leg”, and 1 point for “Sensory,” for a total of 10 points [15]. The Manual Muscle Test (right/left) results were as follows: deltoid 5/0, biceps 5/0, extensor carpi radialis longus 5/0, triceps 5/0, flexor digitorum profundus 5/0, abductor pollicis minor 5/0, iliopsoas 4/2, quadriceps 4/3, tibialis anterior 4/0, extensor digitorum longus 4/0, and triceps muscle of calf 4/0 [16]. Range of motion was −5° in the left knee joint during extension and 5° in the left ankle joint during dorsiflexion, with no obvious restrictions in the other joints. Sensory examination revealed mild blunting of the superficial and deep senses. However, no new acute neurological deficits appeared in the patient. In addition, PS, functional independence measure (FIM), cardiopulmonary exercise testing (CPET), the 6-Minute Walk Test (6 MWT), the 10-m walking test (10 MWT), hand-held dynamometer (HHD) assessments, and body weight were also evaluated. The FIM is an independent rating scale for ADLs, consisting of 13 motor and 5 cognitive items, each of which is rated on a scale of 1 to 7 points, with higher scores indicating better ADL ability. The maximum score is 91 for motor items and 35 for cognitive items, with a maximum total score of 126 [17]. CPET is a well-established test for exercise tolerance that analyzes exhaled gases [18]. In this case, measurements were taken with a bicycle ergometer exercise load and an AERO AE300S (MINATO MEDICAL SCIENCE CO., LTD.) instrument. Exhaled gas analysis was performed using the breath-by-breath method, and peak VO_2_ max was measured. The 6 MWT measured a 6-min walking distance with maximal effort, as indicated by the American Thoracic Society [19]. For the 10 MWT, the shortest time to walk 10 m was measured three times, and the average value was calculated [20]. For the 6 MWT and 10 MWT, the patient walked with a cane while wearing a lower limb orthosis. To determine maximum knee-joint extension muscle strength on the left and right sides, HHD was measured three times using μ-tas F-1 (ANIMA Co., Tokyo, Japan) and the average value was calculated [21]. The patient was given light assistance due to the risk of falling. Results obtained at the time of examination in the Department of Rehabilitation are shown in Table 2 and Table 3. The PS was 3, the total FIM score was 97 points, the motor score was 62 points, and the cognitive score was 35 points. The ADLs that showed particularly low ability were lower body dressing, toileting, bed-to-chair transfer, toilet transfer, shower transfer, locomotion, stairs, and other movements that mainly affected lower extremity function. VO_2_ max in CPET was 821 mL/min and 14.1 mL/kg/min; 6 MWT was 105 m; and 10 MWT was 33.2 s. Lower limb muscle strength in the HHD assessment was 80 N in the right leg and 42 N in the left leg.

The patient’s PS was thought to have declined due to the loss of muscle strength in the healthy side of the lower limb and the impaired mobility caused by severe left hemiplegia. However, since the patient had previously been able to walk with a cane, the paralyzed leg was probably strongly affected by muscle weakness due to disuse, which was also apparent in the healthy leg muscles; therefore, it was judged that there was sufficient room for improvement through rehabilitation, and rehabilitation was started on the same day. The main objective of rehabilitation was to improve PS by increasing muscle strength in both lower limbs and improving ADL. In addition, since the patient’s VO_2_ max was 14.1 mL/kg/min, which is low enough for a concerning high risk of postoperative complications, we aimed to improve VO_2_ max as much as possible before surgery.

**Figure 1:**
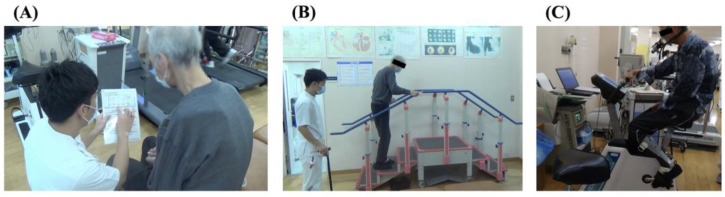
Preoperative rehabilitation. (**A**) Preoperative rehabilitation guidance was provided using a pamphlet prepared by the Department of Rehabilitation. (**B**) Muscle-strengthening exercises included climbing steps and stairs. (**C**) Cardiopulmonary exercise testing.

This hospital provides high-intensity, long-duration rehabilitation therapy tailored to the patient’s general condition [22,23]. After admission, patients undergo approximately 2 h and 30 min of preoperative rehabilitation twice a day: once in the morning and once in the afternoon. The preoperative rehabilitation period lasts 22 days and includes 44 sessions of rehabilitation. During the initial rehabilitation, we use a pamphlet prepared by the Department of Rehabilitation Medicine to instruct the patient on deep diaphragmatic breathing, efficient coughing and huffing with intense contraction of the abdominal muscles, as well as the rehabilitation process from preoperative to postoperative discharge (Figure 1A). Muscle strengthening exercises include squats (300 times/session), calf raises (100 times/session), and step ascents and descents using a 20-cm step (100 times/session) (Figure 1B). Aerobic exercise consists of bicycle ergometer exercise (30 min/session) and hand ergometer exercise (20 min/session). The exercise load is set at 70–80% load using the heart rate (HR) reserve method, with peak HR measured by CPET (Figure 1C). The physical therapist mainly directs repetitive gait training, stair climbing, and ADL training. Muscle strength training and aerobic exercises are also supervised by the physical therapist, but most of these are performed by independent trainers.

The results of the 22-day preoperative rehabilitation are shown in Table 2 and Table 3. The FIM showed improvement in all items that had previously been declining, with a total score of 117. In addition, the patient became independent when walking with a cane and in all personal activities in the hospital. The patient’s PS improved to 2 due to his increased independence in ADLs in the hospital. CPET results showed an increase in VO_2_ max of 959 mL/min and 16.6 mL/kg/min. Walking ability tests were performed without assistance from a physical therapist, as the patient was walking independently with a cane and wearing a brace. Consequently, his 6 MWT distance extended to 125 m, and his 10 MWT time decreased to 30.9 s. The HHD results also increased to 120 N and 82 N on the right and left sides, respectively. Due to this improvement in physical function, the indication for surgery was reconsidered at a preoperative conference of the Gastrointestinal Surgery Department. Therefore, the patient was judged to be operable, and surgery was subsequently performed.

The surgical techniques performed were: thoracoscopic subtotal esophagectomy, thoracoscopic gastric tube creation, two-region lymphatic dissection, and reconstruction of the posterior sternal pathway gastric tube. The operation time was 8 h and 21 min, and the operative blood loss was 45 mL. The postoperative diagnosis was esophageal squamous cell carcinoma (T3, N1, M0, and stage III). Postoperatively, the patient was admitted to the intensive care unit for management.

**Figure 2:**
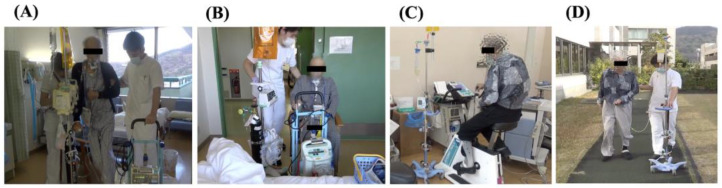
Postoperative rehabilitation. (**A**) Walking down the hallway for the first time at 11:30 am after discharge from the intensive care unit (postoperative day 1). (**B**) The patient was instructed to spend as much time as possible during the day in a sitting-chair position (6 h/day) outside of rehabilitation (postoperative day 1). (**C**) The patient resumes bicycle ergometer exercise while on continuous infusion with an enterostomy (postoperative day 7). (**D**) The patient is able to walk outdoors unassisted before discharge (postoperative day 26).

On the first postoperative day (POD), at 8:00 a.m. the patient was extubated from the ventilator and at 11:00 a.m. transferred to the general ward. Mobilization was performed at 11:30 a.m., and gait training began with assistance in cooperation with a nurse. Supplemental oxygen therapy was administered using a mask at a flow rate of 10 L/min. The patient had a right chest drain, a cervical drain, and a J-tube. (Figure 2A). The patient was instructed to spend as much time as possible (6 h/day) in a chair-sitting position outside of daytime rehabilitation (Figure 2B). After POD 5, the training location was moved from the ward to the rehabilitation room. While training in the rehabilitation room, the frequency and distance of gait training increased appropriately. Low-intensity muscle strength training was initiated. Supplemental oxygen therapy was discontinued for POD 7. Bicycle ergometer exercises were added to the training program (Figure 2C), and the muscle strength training load was increased. The patient was eventually able to walk outdoors unassisted (Figure 2D) and was discharged home on POD 26. The results of the evaluation at discharge are shown in Table 2 and Table 3. At discharge, the patient had a PS of 2 and a total FIM score of 117, and he maintained an ADL ability equivalent to that of the day before surgery. CPET results were VO_2_ max of 980 mL/min and 17.8 mL/kg/min; 6 MWT was 128 m; 10 MWT was 26 s, showing improvement in comparison to his results on the day before surgery. There was no dysarthria or dysphagia after the CVA onset. Postoperatively, there was no hoarseness or obvious dysphagia; a jelly diet was started on POD 11. On POD 15, the diet was changed to a soft, bite-sized diet. In addition, there were no adverse events during rehabilitation from the preoperative period to discharge, and no postoperative complications occurred. After being discharged from the hospital, the patient was followed up by a gastroenterological surgeon once a month; there has been no recurrence, and the patient is living independently.

In accordance with the local legislations and institutional requirements, ethical review and approval were not required for this case report. In order to publish any potentially identifiable images or data included in this article, a written informed consent was obtained from the patient and his family. This study conforms to all case report guidelines and reports the required information accordingly.

## 3. Discussion

Most of previous reports on preoperative rehabilitation for patients undergoing esophageal cancer surgery have focused on preventing postoperative respiratory complications, atelectasis, and shortening hospital stay duration [6,7,8,9,10]. This case report is the first to detail the preoperative-to-postoperative course and physical training of a patient with esophageal cancer. This patient was deemed ineligible for surgery due to a gradual decline in PS over the course of 8 years after a stroke, but became eligible for surgery after 3 weeks of intensive preoperative rehabilitation and was then discharged home.

A previous study reported a preoperative rehabilitation program for the prevention of pulmonary complications consisting of respiratory rehabilitation, including respiratory muscle and thoracic stretching to increase pulmonary compliance, deep breathing and abdominal breathing training, sputum training, muscle strength training using weights, and 20 min of bicycle ergometer exercise, for approximately 60 min per day, respectively [6]. In other reports, training consisted of respiratory rehabilitation, upper and lower limb and abdominal muscle strength training, and 15 min of ergometer exercise, for 40–60 min per day, respectively [7]. Akiyama et al. employed 20–30 min of ergometer exercise at 60–70% of maximum HR, and 20 sets of 10–15 squatting exercises twice a day for 7 days [9]. Since rehabilitation in this case aimed to improve ADL and PS, the patient was instructed to perform 300 squats, 100 calf raises, 100 step ascents and descents, 30 min of lower limb ergometer exercise at 70–80% of HR reserve, 20 min of upper limb ergometer exercise, and one set of ADL training, including gait training, in the morning and afternoon, for a total of approximately 5 h per day of long, high-intensity rehabilitation. Previous studies on the effects of rehabilitation on patients with chronic cerebrovascular disease have shown that 60 min of physical therapy, including gait training 4–5 times a week for 4 weeks, increases muscle strength in both paralyzed and nonparalyzed limbs [24]. Endurance exercise, balance training, and resistance exercise were performed for 60 min, three times a week for 19 weeks, and improved walking ability, walking speed, 6-min walking distance, and muscle strength, especially in the paralyzed side of the lower limb [25]. In a meta-analysis of acute, subacute, and chronic stroke patients, peak VO_2_, peak workload, walking speed, and walking endurance increased with only 20–40 min of endurance exercise 3–5 times per week for 10 weeks [26]. Zaiki et al. found significant improvement in FIM in patients with chronic cerebrovascular disease who performed 75 min of rehabilitation twice a week for 1 year but no improvement in patients who performed only 50 min of rehabilitation, suggesting the importance of rehabilitation duration [27]. Although the duration of preoperative rehabilitation was 3 weeks in this report, which was shorter than in previous studies, it is possible that the longer daily rehabilitation time and addition of high-intensity loads improved ADL earlier. In addition, the repetitive training for gait and mobility impairments directed by the physical therapist was thought to have contributed significantly to the improvement of ADL ability and PS.

A review by Guinan et al. [28] indicated that early mobilization after surgery in patients with esophageal cancer is important for enhancing recovery after surgery to maximize the increase in lung function, prevent gastroesophageal reflux, and prevent postoperative complications such as atelectasis due to basal ventilation/perfusion matching. Many reports also recommend frequent early postoperative activity starting on POD 1, with a wide variety of specific recommendations, but most agree that these activities should include sitting out of bed for at least 2 h and short walks (10 m/100–200 feet). Haines et al. [29] reported that the type of surgical incision and time to early mobilization were independently associated with the occurrence of postoperative pulmonary complications and that patients who could not get out of bed on POD 1 were 3.0 times (95% confidence interval 1.2–8.0) more likely to have such complications. In addition, preoperative VO_2_ max correlated strongly with survival in patients with esophageal and gastric cancer [30], and preoperative peak VO_2_ was found to be higher in patients without postoperative complications than in those with postoperative complications [31]. In this study, the patient was discharged home without any postoperative complications, and his ADL ability post-discharge has improved compared to that preoperatively; in addition, he can move around independently and perform personal activities without the assistance of his family. The patient was trained to walk more than 50 m with assistance the day after surgery with the assistance of nurses and was encouraged to sit in a chair for 6 h/day after walking to reduce the amount of time he spent lying in bed. Moreover, the patient’s VO_2_ max was improved by preoperative rehabilitation, which may have helped prevent complications. In addition to the preoperative improvement in ADL ability, the patient’s general condition did not deteriorate, and he did not experience any complications; this was due to the implementation of muscle strength training and aerobic exercises, as well as the gradual increase in exercise load, which enabled discharge home with better ADL ability than before preoperative rehabilitation had begun.

This study reported one case only. The cerebrovascular disease patients have a wide range of sequelae and a varying disease severity; thus, it is difficult to generalize the content and course of rehabilitation before and after surgery. In some cases, two-stage surgery may be indicated. However, this report describes a patient with esophageal cancer who was judged to be ineligible for surgery due to low activity and tolerance for surgery but became operable after 3 weeks of intensive preoperative rehabilitation. The content and course of rehabilitation in this case may provide valuable information for patients with esophageal cancer and low activity.

## 4. Conclusions

In some cases of esophageal cancer, providing 3 weeks of high-intensity prolonged preoperative rehabilitation to patients that are ineligible for surgery can improve ADL and PS to the point of surgical indication. Therefore, cancer patients who are judged to be inoperable owing to low activity may benefit from intensive rehabilitation in a hospital before deciding on a treatment plan.

## Figures and Tables

**Table 1:** Performance Status Scales.

Score	Description
0	Fully active, able to carry on all pre-disease performance without restriction.
1	Restricted in physically strenuous activity, but ambulatory and capable of performing a light or sedentary work (e.g., light housework or office work).
2	Ambulatory and capable of all selfcare but unable to carry out any work activities. Up and about more than 50% of waking hours.
3	Capable of only limited selfcare, confined to a bed or a chair for more than 50% of their waking hours.
4	Completely disabled. Cannot carry out any selfcare. Totally confined to a bed or a chair.

This table was modified by the authors, based on a reference [2] (in Japanese).

**Table 2:** Medical records of patients in the present case study.

	On Admission	Day before Surgery	At Discharge
PS	3	2	2
Total FIM	97	117	117
Motor subscale	62	82	82
Cognition subscale	35	35	35
Body weight (kg)	58.0	57.5	55.0
CPET			
VO_2_ max (mL/min)	821.0	959.0	980.0
VO_2_ max (mL/Kg/min)	14.1	16.6	17.8
6 MWT (m)	105.0	125.0	128.0
10 MWT (s)	33.2	30.9	26.0
HHD			
Right (N)	80.0	120.0	120.0
Left (N)	42.0	82.0	82.0

PS, Performance Status; FIM, Functional Independence Measure; CPET, Cardiopulmonary Exercise Testing; 6 MWT, 6-min walk test; 10 MWT, 10-m walk test; HHD, Hand-held dynamometer.

**Table 3:** Changes in FIM during each period.

	On Admission	Day before Surgery	At Discharge
FIM of the total	97	117	117
Motor subscale	62	82.0	82.0
Eating	6	7	7
Grooming	5	7	7
Bathing	5	6	6
Upper body dressing	5	6	6
Lower body dressing	4	6	6
Toileting	4	6	6
Bladder management	7	7	7
Bowel management	7	7	7
Bed to chair transfer	4	6	6
Toilet transfer	4	6	6
Shower transfer	4	6	6
Locomotion (ambulatory or wheelchair level)	4	6	6
Stairs	3	6	6
Cognition subscale	35	35	35
Cognitive comprehension	7	7	7
Expression	7	7	7
Social interaction	7	7	7
Problem solving	7	7	7
Memory	7	7	7

FIM, functional independence measure. FIM tallies 18 items for daily living, which are graded on a 7-point scale: 1, total assistance; 2, maximal assistance; 3, moderate assistance; 4, minimal contact assistance; 5, supervision or set-up; 6, modified independence; and 7, complete independence. The full score is 126 points, with a minimum score of 18 points. The higher the score, the more independent the patient is.

## Data Availability

The authors’ raw data supporting the conclusions of this article will be made available upon reasonable request.
